# The Contribution of BaTiO_3_ to the Stability Improvement of Ethylene–Propylene–Diene Rubber: Part I—Pristine Filler

**DOI:** 10.3390/polym15092190

**Published:** 2023-05-05

**Authors:** Tunde Borbath, Nicoleta Nicula, Traian Zaharescu, Istvan Borbath, Tiberiu Francisc Boros

**Affiliations:** 1 ROSEAL SA, 5 A Nicolae Bălcescu, Odorheiu Secuiesc, 535600 Harghita, Romania; borbath.tunde@gmail.com (T.B.);; 2 INCDIE ICPE CA, 313 Splaiul Unirii, 030138 Bucharest, Romania

**Keywords:** ethylene–propylene–diene rubber-based composites, barium titanate as oxidation protector filler, radiation stability, chemiluminescence, mechanical testing, antifungal strength

## Abstract

This study presents the functional effects of BaTiO_3_ powder loaded in ethylene–propylene–diene rubber (EPDM) in three concentrations: 0, 1, and 2.5 phr. The characterization of mechanical properties, oxidation strength, and biological vulnerability is achieved on these materials subjected to an accelerated degradation stimulated by their γ-irradiation at 50 and 100 kGy. The thermal performances of these materials are improved when the content of filler becomes higher. The results obtained by chemiluminescence, FTIR-ATR, and mechanical testing indicate that the loading of 2.5 phr is the most proper composition that resists for a long time after it is γ-irradiated at a high dose. If the oxidation starts at 176 °C in the pristine polymer, it becomes significant at 188 and 210 °C in the case of composites containing 1 and 2.5 phr of filler, respectively. The radiation treatment induces a significant stability improvement measured by the enlargement of temperature range by more than 1.5 times, which explains the durability growth for the radiation-processed studied composites. The extension of the stability period is also based on the interaction between degrading polymer substrate and particle surface in the composite richest in titanate fraction when the exposure is 100 kGy was analyzed. The mechanical testing as well as the FTIR investigation clearly delimits the positive effects of carbon black on the functionality of EPDM/BaTiO_3_ composites. The contribution of carbon black is a defining feature of the studied composites based on the nucleation of the host matrix by which the polymer properties are effectively ameliorated.

## 1. Introduction

The production of various polymers and their composite items requires detailed and accurate investigations of the functional characteristics, such as mechanical strengths, long-term stability, and biological solution to the fungi attack that describe complementary global durability under hazardous conditions [1]. The analysis of operational performance is the main goal of material qualification when the technical products are destined for nuclear power plants [2]. For the keystone analysis of radiation effects on polymers, a comprehensive book chapter was published [3]. The most important aspect that reveals the radiochemical stability and the durability of polymer materials is the value of the ratio between the radiochemical yields of scission and crosslinking, G(S)/G(X). Its superunitary value, resulting from the three times higher ethylene segments with respect to the propylene content, is proof for an appropriate radiation processing or long-term operation in nuclear applications. The individual values of G(S) and G(X) for ethylene–propylene–diene rubber (EPDM) are placed between the material characteristics of polyethylene (G(X) = 0.8–1.1 and (G(S) = 0.4–0.5) and polypropylene (G(X) = 0.8–1.1 and (G(S) = 0.4–0.5) [3]. The consumption of diene (ethylene–norbornene) with a radiochemical yield of 32.1 [4] and the formation of various oxygenated degradation products (ketones—G = 13.9, acids—G = 4.4, alcohols—G = 4.1 and peroxides—G = 0.3) depict the behavior of this elastomer under the action of high energy radiation when the protector is not technologically added.

The thermal and radiochemical stabilization effects in the studied elastomer correspond to the technical requirements for various applications such as sealants, vibration buffers, automotive items, medical wear, corrosion protection layers, hydrophobic impregnations, and shoe soles. There are many alternatives through which EPDM may gain improved durability by the addition of phenolic antioxidants [5,6,7], inorganic fillers [8,9,10], and wood wastes [11]. If the evolution of radiation-processed EPDM was already reported [12], the stabilization mechanisms promoted by the inorganic materials based on the surface interactions through which the free radicals born during the oxidative degradation are still elaborating [13].

The inhibition of degradation is effectively accomplished by the appropriate compounds, which are able to break the oxidation chain. On the propagation stage, the conversion of peroxyl radicals into final stabile products would be efficiently interrupted if the filler does not act on the free radicals by bonding or the active component exceeds a certain critical threshold. The hazardous conditions existing in nuclear power plants require a certain advanced stability by which the rubber products are able to resist with proper functional parameters between the successive maintenance stops [14]. Under various circumstances, the nature and loading degree of filler [15], as well as the content of diene composing the elastomer structure [16], are determining factors that characterize the material strength against oxidation [17]. The high-performance materials that are radio-oxidation resistant may be obtained either by the incorporation of another suitable polymer to promote crosslinking [18,19], the addition of a crosslinker [20], or the compatibilization of an efficient filler [21]. Accordingly, high-tech materials may be produced by the inspired combination between the compositional features and the foreseen technological parameters [22].

**Figure 1:**
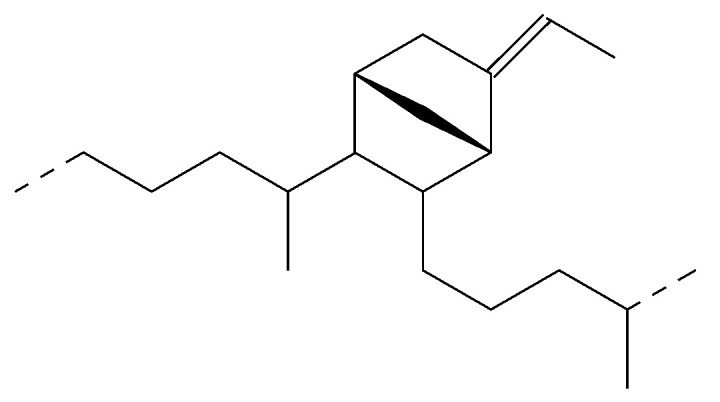
The structure of ethylene-propylene-diene-monomer.

Crosslinked EPDM is a suitable material for several operation areas where the degradation factors do not alter the functional features. The technical concern of stability characteristics is related to the double role of the contained diene: a connecting element between the molecular chains (Figure 1) and the weaker spot that is facilely broken.

During the exposure of this polymer to a certain γ-dose not greater than and especially less than 100 kGy, the scission of norbornene and the bonds of tertiary carbon atoms from propylene moieties create radicals that are involved in crosslinking and oxidation [4,23]. The presence of any oxidation protector increases the crosslinking degree due to the turning of radicals onto the reaction with another analogous fragment [24].

The stability characterization of EPDM is a good opportunity for the presentation of various faces of material qualification when different approaches reveal the peculiar features of a product in connection with its structural understanding. The thermal analysis assay [25], the improvement of ablation resistance [26], the explanation of dielectric properties under various environmental conditions [27], the preparation of new structural materials by the blending EPDM with unlike products such as NBR [28], PP and PA6 [29] or Kevlar fibers [30], the increase in crosslinking degree [31,32,33], the anti-migration effects of graphene oxide [34], and many other practical aspects asked by the market are the study themes that become important for certain applications. One interesting aspect that is related to the valorization of EPDM by converting it into composites or blends is the recycling of its wastes [35,36], the main reason that it may be considered as an example for many other polymer materials.

The modification of the functional performances of EPDM-based products by the addition of an advisable blending component represents an attractive goal on which the radiation processing may create new material structures with enlarged application areas. The investigations on the stability effects of aluminum trioxide on fire retardancy [37], improvement of mechanical characteristics required by electrical applications [38], assay on radiation shielding for source depositing [39], changes in electrical properties of cables for nuclear power plants [40], and applications in the production of commodities [41] are the topics investigated, by which the selected compositions may offer the desired versions of focused interest.

The present assay is addressed to the manufacturers on long-life polymer items, which may resist certain energetic efforts. As it was previously demonstrated [9], the presence of titanate filler is a good solution for the production of high-tech polymer composites. If this early manuscript concerns the pristine barium titanate, the second part of the study will report the stabilization effects brought about by doped barium titanates, which act more efficiently on the degradation rate of EPDM by radical scavenging.

## 2. Materials and Methods

In the present paper, the composite materials based on EPDM are tested for their application in various environments that promote accelerated oxidation.

### 2.1. Materials

The polymer matrix was manufactured by ethylene–propylene–diene terpolymer (EPDM) used in the DSM Elastomers (Heerlen, The Netherlands) as KELTAN 8340. The filler, barium titanate, was purchased from Thermo Scientific (Shandong Deshang Chemical Co., Ltd., Jining, China). The pristine polymer material contained ethylene and propylene moieties in the proportion of 3:1 and 5 wt% 5-ethylidene 2-norbornene (EBN). While the preparation of sheets for the mechanical testing of samples was performed according to an appropriate procedure, the thermal and radiation stabilities were checked on the thin pellicles obtained by the solvent removal. This raw polymer was not subjected to any purification process before the present investigations. Barium titanate has the following characteristics: purity > 99% and an average particle size of 300 μm.

### 2.2. Sample Preparation

For mechanical testing, the plate samples were obtained by means of the thermal pressing process achieved at 180 °C for 15 min. The equipment was produced by Nicovala (Sighisoara, Romania), whose features (plate dimensions: 400 mm × 450 mm, charge: 160 tf, electrical heating) allow the optimal preparation of EPDM plates. The vulcanization pressure was established at 100 bars. From these plates, standard dumbbells according to ISO 527 were obtained. The black samples contain a significant amount of carbon content (36 phr).

For chemiluminescence investigation, thin films were obtained by solvent removal from aluminum round caps (diameter 5 mm) where the liquids were poured. Three concentrations of barium titanate (0, 1, and 2.5 phr) were prepared by the addition of appropriate amounts of powder followed by vigorous shaking for homogenization. Finally, when the dry samples were obtained, they were weighted for the determination of specimen masses. These mass values were used for the normalization of measured CL intensities to the standard unit allowing reliable results for their comparison. Photon counting is achieved by the connection between the experimental device and the attached computer. The specific program for the addition of counting information allows the conversion of results into the Origin representations, which are presented herein.

### 2.3. γ-Irradiation

The radiation treatment was achieved in air at room temperature in an Ob Servo Sanguis irradiation machine (Budapest, Hungary) provided with a ^60^Co source. Three doses were selected (0, 50, and 100 kGy) for the radiation processing of specimens. The dose rate was 0.5 kGy h^−1^. All measurements were performed immediately after the end of exposures, avoiding any alteration of results due to the decay of short-life radicals.

### 2.4. Characterization of Filler

Several attempts for the identification of any differences between the XRD, Raman, and FTIR spectra as well as the SEM images of crystalline pristine and γ-irradiated (100 kGy) barium titanate powders failed. The overlapping of the recorded results did not allow the detection of any radiation effect on this inorganic phase. These details prove that there were no radiolysis effects on the inorganic blending component.

### 2.5. Characterization of Composites

#### 2.5.1. Mechanical Testing

Mechanical testing was achieved using the ZMR 250 equipment (VEB THURINGER, Raunestein, Germany) applying the testing standard ISO 37/2012. The evaluation of Shore A hardness was based on ISO 7619/1 (2011) with testing unit equipment purchased from STENDAL, Stendal, Germany).

#### 2.5.2. Chemiluminescence (CL)

The stability assay was achieved by means of a LUMIPOL 3 device produced by the Institute of Polymers, Slovak Academy of Science, Bratislava (Slovakia). The two available investigation procedures, isothermal and nonisothermal, were used for the complementary assay. For nonisothermal determinations, there were four selected heating rates: 5, 10, 15, and 20 °C min^−1^. The isothermal measurements were achieved at 160, 170, and 180 °C. The CL spectra were electronically transferred from the measurement device onto the computing system, which allows the graphical approach.

#### 2.5.3. Spectral Assay by FTIR-ATR

A Fourier transform infrared (FTIR) spectrometer (Interspectrum, INTERSPEC 200-X, Tõravere, Estonia) equipped with a device for attenuated total reflectance (ATR) was used in the wavenumber range between 4000 cm^−1^–750 cm^−1^ at room temperature for evaluation of samples spectra. Each ATR–FTIR spectrum is an average of over 10 scans, using air as a reference, and 4 cm^−1^ as the nominal spectral resolution.

#### 2.5.4. Antifungal Properties

The antifungal properties of the samples consisting of EPDM and barium titanate in various proportions were inspected according to ASTM-G21-09 standard [42]. Briefly, small square samples (2.5 cm × 2.5 cm) with a smooth surface and free of defects were placed in Petri dishes containing a layer of Chapex-Dox agar. A 0.1 mL of fungal spore suspension (consisting of a mixture of *Aspergillus brasiliensis*—ATCC 9642, *Penicillium funiculosum*—ATCC 11797, *Chaetomium globosum*—ATCC 6205, *Trichoderma virens*—ATCC 9645, and *Aureobasidium pullulans*—ATCC 15233) was poured onto the sample surface. These samples were incubated for 28 days at 27 ± 2 °C and 95% relative humidity. After the incubation period, the samples were visually evaluated for fungal growth. The extent of growth was rated on a scale of 0–4. Number 0 indicates the lack of growth, while Number 4 indicates intense growth covering the entire sample surface. The evaluation of fungal growth extent is useful for the nomination of inspected materials that are “resistant” or “non-resistant” products. Materials with a rating of 0 or 1 are considered resistant materials, while the other ones presenting a rating of 2, 3, or 4 are considered non-resistant products. The assay was conducted in triplicates.

## 3. Results

The thermal stability is directly related to the breadth of degradation that is initiated by the formation of peroxyl radicals [4] The oxidation is propagated by the further reactions of RO_2_**^.^**, whose generating rate depends on the free radical abundance and the diffusion rate of molecular oxygen. In the case of EPDM, the most vulnerable spots are the position of methyl in the polypropylene moieties and the unsaturation of diene (EBN) [43]. The evolution of oxidation in the presence of stabilizers during the radiation processing of EPDM products occurs differently, because the antioxidants may turn on the mechanisms in peculiar ways [44]. Even though the γ-irradiation is an accelerated process of degradation, it may be delayed in two different manners: crosslinking [45] and/or stabilization [46].

The progress of oxidative degradation caused by accidental or current energy depositing onto polymeric items may be slowed down by the effect of inorganic structures and determines the diffusion and retention of free radicals inside the traps of stabilizer [9,47]. The delay of aging is always correlated with the stabilization efficiency that characterizes the strength of scavenging and the size of available free space inside the structured oxidation protector [48]. The γ-processing of polymers that causes profound damage to macromolecules may be judged as a proper way of material improvement when the stabilization action of filler is accompanied by radiation crosslinking [49]. In the case of EPDM, this assumption is valid, because this polymer belongs to the polymer class of radiation crosslinking materials [50].

### 3.1. Chemiluminescence

The emission of photons during polymer oxidation from the reacting entities, according to the deexcitation of the triplet state of ketones onto the background, allows the observation of the evolution of degradation. It is obvious that the progress of oxidation involves the counting of the quantum number that is proportional to the concentration of peroxyl radicals [51]. The shapes of CL curves offer real indications concerning the fate of radicals and their decay in relation to the presence of oxidation protectors [52].

#### 3.1.1. Nonisothermal Chemiluminescence

Certain formulations of material composition lead to peculiar thermal behavior, which is the overall result involving the contributions of blended components. Accordingly, the temperature when the oxidation starts is determined by the easiness of splitting of the weakest existing bond. The influence of composition on the thermal stability of EPDM is demonstrated by the evolution of oxidation with and without carbon black (Figure S1). Even though the addition of carbon black initiates a crosslinking effect [53], it also provides an effect of stabilization. This feature was previously reported [54,55]. Fortunately, photon counting is possible with both groups of samples (with and without carbon black). However, the application of isothermal determinations as the measurement procedure failed with the black samples, this is because the long-time counting involves the self-absorption of CL photons over the first part of the experiment.

**Figure 2:**
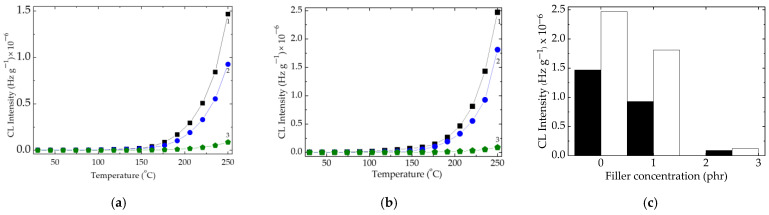
Nonisothermal CL spectra recorded on (**a**) polymer with carbon black and (**b**) polymer without carbon black. Filler concentration: (1) 0 phr, (2) 1 phr, and (3) 2.5 phr. Heating rate: 15 °C min^−1^. (**c**) Histogram illustrating the CL intensities at 250 °C. (black) polymer with carbon black; (white) polymer without carbon black.

The nonisothermal CL measurements of the EPDM-based composites reveal the influence of filler concentration on the progress of oxidation when the testing temperature monotonically increases (Figure 2). The evident effect is the increase of starting oxidation temperature as the filler loading becomes higher. The presence of carbon black in the sample formulations causes a diminution of CL emission. It may be assumed that the oxidation is inhibited by carbon black as it was also previously reported [38].

The increase in the filler loading determines an evident improvement in the polymer stability, which reaches a good degree when the barium titanate has a concentration of 2.5 phr (Figure 2). This behavior is in good agreement with the previous results obtained by the preparation of nanocomposites [56]. The comparison of CL intensities measured at 250 °C provides obvious proof for the contribution of carbon black on the material stabilization during thermal oxidation.

**Figure 3:**
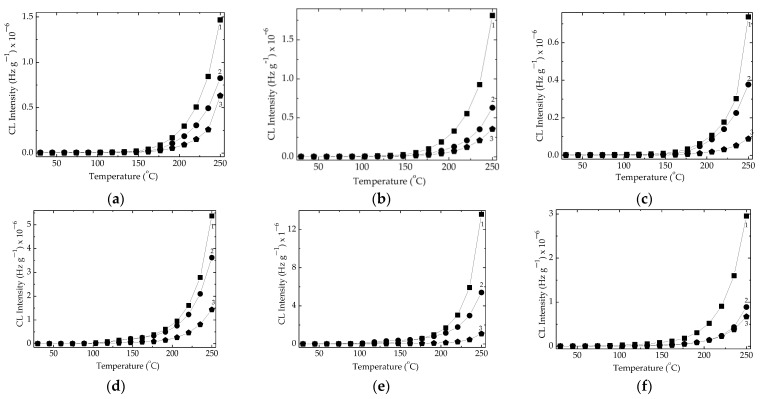
Nonisothermal CL spectra recorded on composite EPDM/BaTiO_3_ probes; filler contents: (1) 0 phr; (2) 1 phr; (3) 2.5 phr. Samples with carbon black: (**a**) D 0 kGy, (**b**) D 50 kGy (**c**) D 100 kGy. Samples without carbon black: (**d**) D 0 kGy, (**e**) D 50 kGy, (**f**) D 100 kGy. Heating rate: 15 °C min^−1^.

The influence of filler concentration on the development of oxidation is illustrated in Figure 3. The consequences of γ-irradiation on the progress of oxidation are related to the availability of EPDM for crosslinking, being known that the dose range up to 100 kGy is proper for the increase of its insoluble fraction [18,57]. This trend of amelioration in the polymer stability is more evident at a higher dose (100 kGy) when the involvement of free radicals in the oxidative degradation is really prevented by the presence of inorganic filler. The extension of the stability range of temperature as the fillet content and processing γ-dose growth is a main advantage; therefore, these compositions are recommended as the proper formulations for their applications in degradation-providing environments.

#### 3.1.2. Isothermal Chemiluminescence

**Figure 4:**
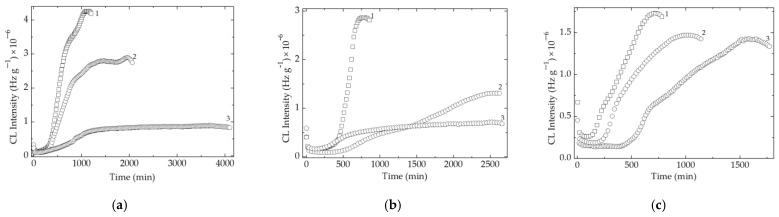
Isothermal CL spectra recorded on composites containing EPDM and BaTiO_3_ without carbon black exposed to various γ-doses. Testing temperature: 170 °C. Filler content: (1) 0 phr, (2) 1 phr, (3) 2.5 phr. (**a**) 0 kGy, (**b**) 50 kGy, (**c**) 100 kGy.

The evolution of degradation at a constant temperature (170 °C) reveals the high stability degree in the presence of barium titanate (Figure 4). The effects on the oxidation rates, the maximum period of degradation, and the height of emission intensity obtained for the investigated EPDM/BaTiO_3_ composites are relevant for the protective action of filler during the accelerated degradation occurring during γ-irradiation. The chemiluminescence measurements on the EPDM-based composite samples containing carbon black prove (Figure S1) that technical items are more resistant when this additive is incorporated.

The unusual long periods of degradation at 170 °C, even at technological γ-irradiation doses (50 and 100 kGy), are reliable evidence that incorporated filler is able to delay oxidation under hard energetic conditions of operation. The amplitudes of CL emission intensities are low for composite formulations with respect to pristine material, which depicts the jointing tightness of molecular fragments withdrawn from their reaction with diffused oxygen. The differences that exist between the values of oxidation rates, maximum Cl intensities, and oxidation induction times are the relevant indices for the scavenging action of filler particles that superficially takes place before the reactions between free radicals and penetrating gaseous oxygen. The progress of aging is efficiently delayed by the interaction between energetic gaps existing in the outer layers of particle surface and the hydrocarbon moieties resulting from bond scissions. For the low irradiation stages (0 and 50 kGy), the local concentration of oxidation intermediates is not too high. Consequently, the pseudoplateau parts of CL curves are obtained, indicating the efficacious ability of filler grains to catch reacting units. They also indicate the lack of desorption. At 100 kGy, the ascendant curves show the generation of high amounts of oxidation initiators. The gaps become highly populated, and an important fraction of radicals are oxidized prior to their structural blocking. The defects existing in the lattice of titanate may also contribute to the behavior amelioration of EPDM-based materials.

### 3.2. Mechanical Testing

The expected behavior regarding the mechanical properties of the studied formulations is based on the correlation between the effect of filler that inhibits oxidative degradation and the contribution of γ-treatment on the yielded crosslinking degree. As it was previously reported [58], EPDM presents a scission yield of 5.2 × 10^−7^ mol J^−1^, by which this polymer provides free radicals for crosslinking according to the mechanism based on radical recombination [48,59]. The structural dependency of crosslink density is the consequence of the involvement of diene, namely 2-ethylidene-5-norbornene [60], whose contribution to the material curing characterizes the modification that occurred in the mechanical properties of EPDM composites. The aging changes undergone in γ-irradiated EPDM are influenced by the existence of filler, which interacts with basic material by the mechanistic modification of the fate of radicals [61]. This approach was also reported, when the ethylene-propylene-diene elastomer material was modified by the presence of lead vanadate [62] or titania [63].

The degradation of plastics occurs as a fragmentation process [64], when the smaller molecular parts composing the processed materials may be separated. The mechanical properties of these studied samples are tightly related to the level of γ-irradiation [43], whose values are the combination between this scission process and a certain degree of crosslinking [65]. The illustration of this overlapping is based on the extended durability that occurs in nuclear power plants [40,66].

**Figure 5:**
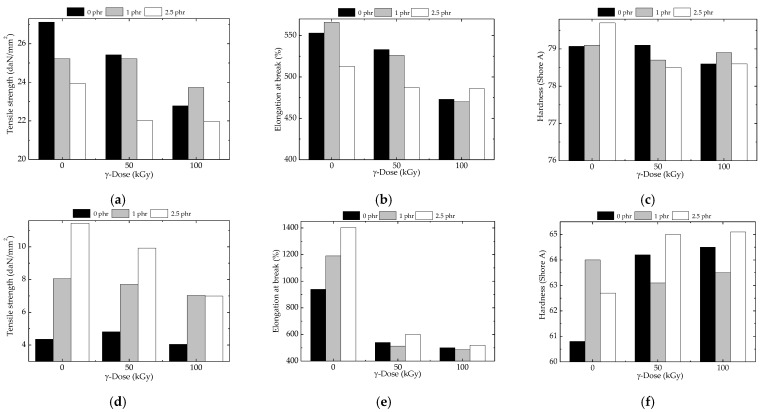
Mechanical behavior of EPDM/BaTiO_3_ composites subjected to γ-irradiation. Samples with carbon black: (**a**) tensile strength, (**b**) elongation at break, (**c**) hardness; samples without carbon black: (**d**) tensile strength, (**e**) elongation at break, (**f**) hardness.

The performances of irradiated composites based on elastomeric materials are the overall effects by which free radicals undergo the reactions that may partially restore the initial characteristics [67]. Because there are differences between the values of mechanical properties (Figure 5), their explanation is based on the evolution of oxidation states whose ways are preceded according to the protective action of filler [68]. The deterioration of the mechanical behavior in EPDM leads to a reduction of molecular mobility by crosslinking. This proof is indicated by the shift of the glass transition relaxation temperature towards higher temperatures.

The reinforcement of EPDM containing relatively low amounts of barium titanate (1 and 2.5 phr) and enough high loading of carbon black (36 phr) is observed after the application of γ-radiation treatment. This means that the fragments that result from the splitting of macromolecules are agglomerated around fillers. They promote nucleation as it occurs in other EPDM composites [69,70]. Of course, the compositions with carbon black are suitable for several applications (O rings, anticorrosive protection, phonic insulations, vibration attenuators, impregnation agents for hydrophobic layers of ceramics, and many other opportunities.

### 3.3. FTIR for the Oxidation of Composites

**Figure 6:**
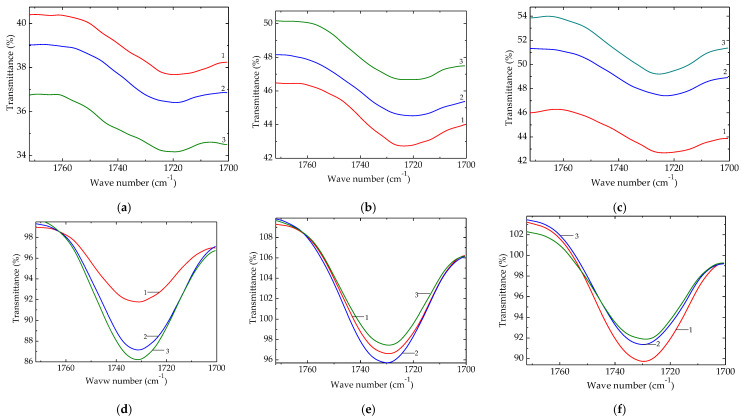
FTIR spectra for EPDM/BaTiO_3_ composites subjected to oxidative degradation under γ-irradiation. Samples with carbon black: (**a**) 0 kGy; (**b**) 50 kGy; (**c**) 100 kGy. Samples free of carbon black: (**d**) 0 kGy; (**e**) 50 kGy; (**f**) 100 kGy. Filler loadings: (1) 0 phr; (2) 1 phr; (3) 2.5 phr.

The course of oxidation progresses during the propagation stage of degradation, when free radicals are attacked by molecular oxygen. It grows the content of oxygenated products, whose evolution may be well illustrated by the modifications occurring in the carbonyl band (Figure 6). There are great differences between the samples containing carbon black and the probes free of it [71].

The modification of absorbance values follows the contributions of various intermediates that produce the oxygenated stabile structures accumulating in the final state of the material. The presence of BaTiO_3_ filler determines a dissimilar modification in the transmittance values for carbonyl vibrations (Figure S2) that provide proof for the protection activity of the inorganic phase by means of its capacity to inactivate radicals in respect to their oxidation. It is well known that EPDM composites are efficiently protected by inorganic fillers [72,73] due to the elemental composition comprising oxygen. However, the structure of the host polymer plays an important role in the concentration, distribution, and inner movement of oxidation promoters [74].

The relative order of peaks (Figure 6) that reflects the involvement of filler particles in polymer aging is relevant for the industrial applications of processed EPDM when the diminution of oxidation rate is the main factor for the extension of material durability. The concentration of 2.5 phr of barium titanate provides evident chemical stability, which is proven by the slower accumulation of carbonyl structures. Though the samples containing carbon black keep a relatively constant oxidation level, the compositions free of carbon black present a relatively linear level of oxidation that signifies an active implication of it in the progress of aging [75]. The interaction between elastomer and CB particle surface was reported [76], demonstrating the formation of slight chemical bonds between the polymer macromolecules and some superficial carbon atoms.

A previously published report on the effects of inorganic fillers, such as carbon black, silica, and nanoclay on ethylene–propylene–diene elastomer [77] stated that the best features are obtained when the inorganic component reaches high amounts. Another proof of the improved strength of EPDM composites is shown by the elastomer fibers containing POSS, a three-dimensional silicon-oxygen polyhedral configuration, which presents a good effect on the extension of durability [78]. The attaching of molecular moieties on the particle surface may be considered an electronegativity action of oxygen atoms contained in titanate molecules. The electrostatic attraction promotes the tight joint of radicals on the inorganic phase, where hydrocarbon fragments are electrically blocked vs the oxidation of polymer.

The studied composites may be considered as good examples for other inorganic compounds that are required for certain special applications.

### 3.4. Resistance against Fungi Actions

Table 1 presents the images of the samples exposed to fungi mixture for 28 days, and Table 2 shows the interpretation of the results according to ASTM G21-09 [42] after 7, 14, 21, and 28 days.

Ethylene–propylene–diene monomer (EPDM) rubber is a synthetic rubber material that has good resistance to heat, weather, and chemicals, which makes it suitable for various industrial and automotive applications. When it comes to microbial behavior, EPDM rubber is generally considered to be resistant to microbial growth. EPDM rubber is non-porous and has a smooth surface, which makes it difficult for microorganisms to adhere to and grow on the surface. Additionally, EPDM rubber is resistant to moisture and water absorption, which further reduces the likelihood of microbial growth [79]. However, it is important to note that under certain conditions, such as exposure to various environment stressors (humidity, moisture, organic compounds, UV and gamma radiations) or their modification with different additives, the microbial behavior of EPDM can be changed, and the microorganisms can grow on EPDM rubber surfaces [80].

While there is some research on the antimicrobial properties of barium titanate (most of them being reported on its nano form), the results are not conclusive, and more research is needed to fully understand its potential as an antimicrobial agent [81]. Some studies have shown that barium titanate nanoparticles can inhibit the growth of certain bacteria and fungi [81,82,83]. One proposed mechanism for this antimicrobial activity is the production of reactive oxygen species (ROS) when the nanoparticles come into contact with water, which can damage the cell membranes of microorganisms [84].

As can be seen from Table 1 and Table 2, all types of non-irradiated EPDM samples show high resistance to fungi (i.e., 0 and 1 ratings). The resistance to fungi for the EPDM samples with barium titanate content seems to be influenced by the irradiation dose, thus at 50 kGy, the resistance to fungi is lower than at 100 kGy for both concentrations of BaTiO_3_, even after the first 7 days of exposure. This can be due either to the higher degree of radio-induced crosslinking of the material at 100 kGy than at 50 kGy, and therefore the enzymatic degradation of the material becomes more difficult, or to a stabilizing effect of the BaTiO_3_ that prevents the degradation of the material. In reality, it is possible to have a synergistic effect of the two types of reticulation-stabilization mechanisms. In the case of EPDM samples with carbon black (CB) and barium titanate, the resistance to fungi is higher than in the samples without CB, even after 28 days of exposure. Although there are not many studies that attest to the antifungal activity of BC, it is possible that it functions as a radio-induced free radical trap or as a decomposer of peroxides to form stable products [85].

This present interpretation is correlated with the results obtained by chemiluminescence and FTIR measurements.

## 4. Discussion

The behavior analysis of all studied formulations reveals the decisive role of fillers that efficiently hinder the oxidation of EPDM as it happens in many polymeric systems with special availabilities like poly (vinylidene fluoride) [86], polyketone [87], polysiloxane [88], polyurethane [89], ultrahigh molecular weight polyethylene [90], and Nafion [91]. The way of polymer stabilization by inorganic compounds becomes open for certain salt structures, which directly act on the breaking degradation chain [9,92]. The initiation of stabilization activity is often related to the capacity of filler or additive for the assistance of structural nonconformity like lattice holes and low electron density [9,93]. The stabilization systems based on structured carbon are appropriate examples for the restructuring of polymer networks [94].

The characterization of the stabilization contribution of barium titanate is suggested by the interaction between the surface of filler particles and available free radicals that may be placed in their neighborhood. A possible explanation is the differences that exist in the electron charge distribution near the lattice gaps. The reliable support is the increase of protection effects as the filler concentration becomes greater (Figure 4). The increase in the temperature values for inspected formulations, when the oxidation effectively starts (onset oxidation temperatures, Figure 2a,b), supports the explanation of protective effects by the superficial concentration of defects. More than that, the decrease in CL intensities either for the samples containing carbon black or for the probes free of it explains the involvement of electronic interaction by which free radicals are scavenged and withdrawn from the degradation chains.

The dissimilar mechanism of stabilization promoted by barium titanate with respect to the classical mechanism ascribed to classical antioxidants concerns the strong physical interactions, which are possible due to the unpaired electron that is present in scission fragments. The exposure of EPDM/BaTiO_3_ composite to the action of γ-rays amplifies the stabilization effects when the irradiation dose is not too high for the initiation of oxidative degradation (Figure 3). The availability of free radicals for their trend of recombination instead of their oxidation is revealed by the evolution of carbonyl vibration (1720 cm^−1^) during the FTIR investigation (Figure 6). The increase in the transmittance values is correlated with the fate of intermediates when they are decayed according to the reactions through which they are combined.

The mechanical properties are also based on the evidence of the anti-aging effects of barium titanate, as it protects oxidation and allows the improvement of material elasticity. The most relevant aspect concerning the amelioration of the mechanical behavior of composites is the dissimilarity between the tensile strength and elongation at break values as well as their evolution by the modification of irradiation dose (Figure 5). Striking differences appear for the sample subjected to 50 kGy, where the increase in received energy reached the gelation dose (15 kGy) [95], and the crosslinking exceeds the degradation. The material properties at 100 kGy become more convenient, but the content of titanate influences the stabilization degree.

Starting with the result analysis from a previous report [96], it is easy to consider that irradiation processing is a welcome procedure for the manufacture of O-rings in the presence of carbon black. However, the favorable course of degradation is found only for the elastomer containing 2.5 phr of barium titanate. Under these circumstances, the nonirradiated materials offer an excellent answer to the fungi attack (Table 1 and Table 2). The radiation processing decreases the resistance at the fungi attack, but it becomes relevant at low filler loading (1 phr). The selection of these formulations for the applications under outdoor conditions, namely electrical cable insulations, is a suitable decision, because these materials present appropriate mechanical properties and oxidation strength, with their irradiation being included in the manufacturing procedure.

The inclusion of titanate into the composition of the EPDM structure is accompanied by the restrictive condition of movement for free radicals. The higher content of titanate determines the hindering of fragments to meet oxygen for their conversion into degradation products. If the initiation stage does not depend on the formulation, the propagation step complicates the mechanism by the overlapping of superficial scavenging and inactivation [97].

The formation of free radicals during the γ-irradiation of EPDM indicates the possibility of crosslinking [18,98]. This feature explains the modification noticed in the values of mechanical properties, which are tightly dependent on the number of intermolecular bridges that appear by irradiation curing. The radiation vulcanization of EPDM [99] may provide a resistant material whose utilization in an energetic hazardous environment would be recommended [100]. The hybrid-structured EPDM compounds with barium titanate allow radiation processing as a suitable version of fabrication for many industrially applied items [101,102].

The presence of some appropriate compounds whose structures recommend them as suitable stabilizers may increase the period of safe usage of EPDM compounds explained by the effective inhibition of oxidation based on strong catching by stable chemical bonds [103,104]. The delay of degradation evaluated by the evolution of FTIR spectroscopy is a fast and convenient effect brought about by certain fillers and additives that extends the material durability without significant influence of mechanical properties. Accordingly, the improvement of barium titanate on the material lifetime is mandatory and required by the optimization of functional properties and the minimizing the destructive effects of degrading stressors. The essential requirement for the inclusion of an oxidation protector is its participation in the overall amelioration of material behavior simultaneously accompanied by the lack of alterations [105].

## 5. Conclusions

The present study reports the conditioned behavior of EPDM substrate by the presence of various amounts of barium titanate particles. The radiation degradation of polymer fraction is delayed by the scavenging action of fillers when the composites are processed by γ-irradiation. The compositional feature individualized by the presence of carbon black powder may be considered as an appropriate characteristic, which leads to high oxidation resistance, good mechanical properties, and slow advancing oxidation states. The significant increase in onset oxidation temperature (the temperature that indicates the start of oxidation) of 10–15 °C for each additional 50 kGy is a main benefit of long-term applications that expends either the operation ranges or the warranty conditions. The proposed composite formulations containing higher titanate loadings may be suitable for the manufacturing of high-tech products capable of keeping their oxidation states at a low level, as it is demonstrated by spectroscopic investigation. The mechanical properties are relevant for safe usage when the content of barium titanate is 2.5 phr. It is well corroborated with the longest oxidation induction times (around 500 min) presented by these γ-processed composites. The chemiluminescence results prove the efficient protection activity of BaTiO_3_ filler when γ-irradiation is applied and the thermal aging in the measuring furnace stimulates lower CL emission from the polymer samples with higher filler concentrations (1 and 2.5 phr). The improved functional characteristics of EPDM composites including barium titanate make the manufacture of a large series of materials possible, which can protect surfaces against corrosion, eliminate fluid loss by perfect and long-term sealing, assure the attenuation of vibrations in the jointing part of bridges or buildings, and protect outdoor devices against the wrong effects of forecast factors.

## Figures and Tables

**Table 1:** Sample images after 28 days of fungal exposure.

Sample	Dose (kGy)		
	0	50	100
EPDM	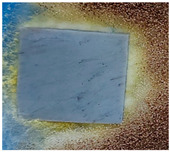	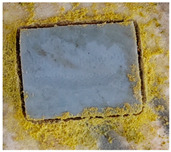	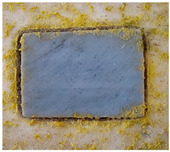
EPDM + 1% BaTiO_3_	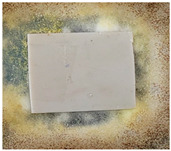	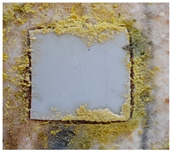	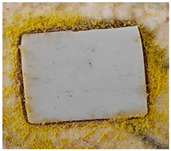
EPDM + 2.5% BaTiO_3_	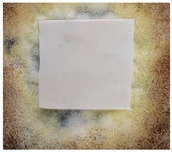	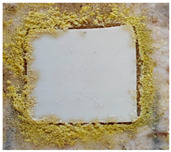	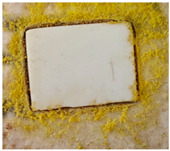
EPDM + carbon black	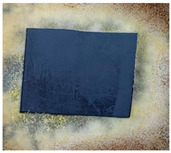	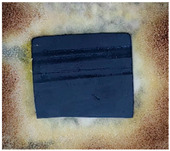	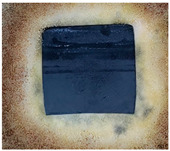
EPDM + 1% BaTiO_3_ + carbon black	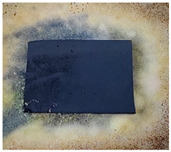	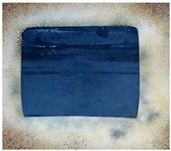	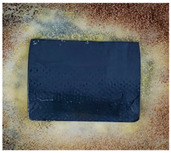
EPDM + 2.5% BaTiO_3_ + carbon black	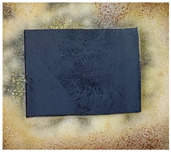	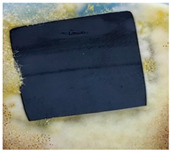	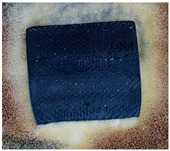

**Table 2:** Sample ratings according to ASTM G21-9.

Sample	7 Days			14 Days			21 Days			28 Days		
	0 kGy	50 kGy	100 kGy	0 kGy	50 kGy	100 kGy	0 kGy	50 kGy	100 kGy	0 kGy	50 kGy	100 kGy
EPDM	0	1	1	0	2	1	0	2	1	1	3	2
EPDM + 1% BaTiO_3_	0	2	1	0	3	1	0	3	1	0	3	2
EPDM + 2.5% BaTiO_3_	0	2	1	0	2	1	0	2	1	0	2	1
EPDM + CB *	0	0	0	0	0	0	0	0	0	0	1	1
EPDM + CB + 1% BaTiO_3_	0	0	0	0	0	0	0	0	0	0	1	1
EPDM + CB + 2.5% BaTiO_3_	0	0	0	0	0	0	0	0	0	0	1	1

* CB means carbon black powder.

## Data Availability

The data presented in this study are available on request from the corresponding authors.

## Supplementary Materials

The following supporting information can be downloaded at: https://www.mdpi.com/article/10.3390/polym15092190/s1, Figure S1: The isothermal CL spectra recorded on pristine EPDM/carbon black samples; Figure S2. The FTIR spectra were recorded on EPDM/BaTiO_3_ composites exposed to a γ-dose of 100 kGy.
