# Supplementary material for: The Contribution of BaTiO3 to the Stability Improvement of Ethylene–Propylene–Diene Rubber: Part I—Pristine Filler
Source: Polymers (Basel). 2023 May 5;15(9):2190. doi: 10.3390/polym15092190 (PMC10181093; doi:10.3390/polym15092190)
Supplement: Supplementary file 1 [file polymers-15-02190-s001.zip › polymers-2307309-supplementary.pdf]

Supplementary materials

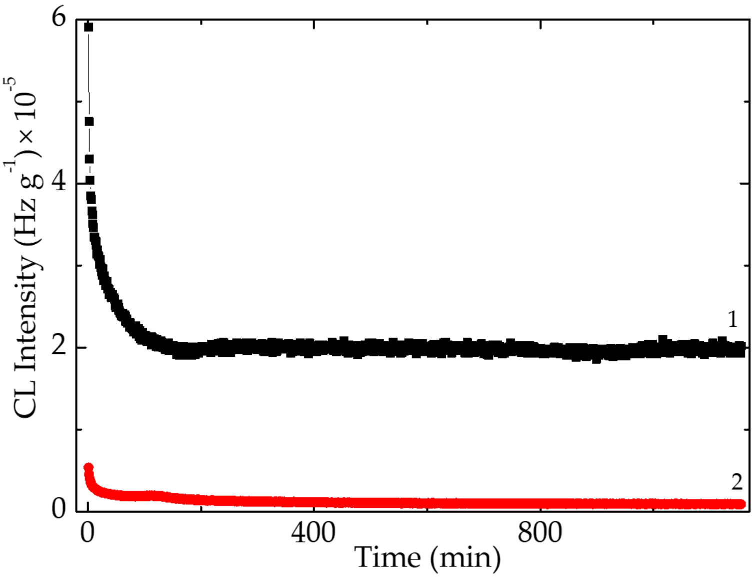

Figure S1. The isothermal CXL spectra recorded on pristine EPDM/carbon black samples.

Temperature: (1) 180 °C, (2) 170 °C.

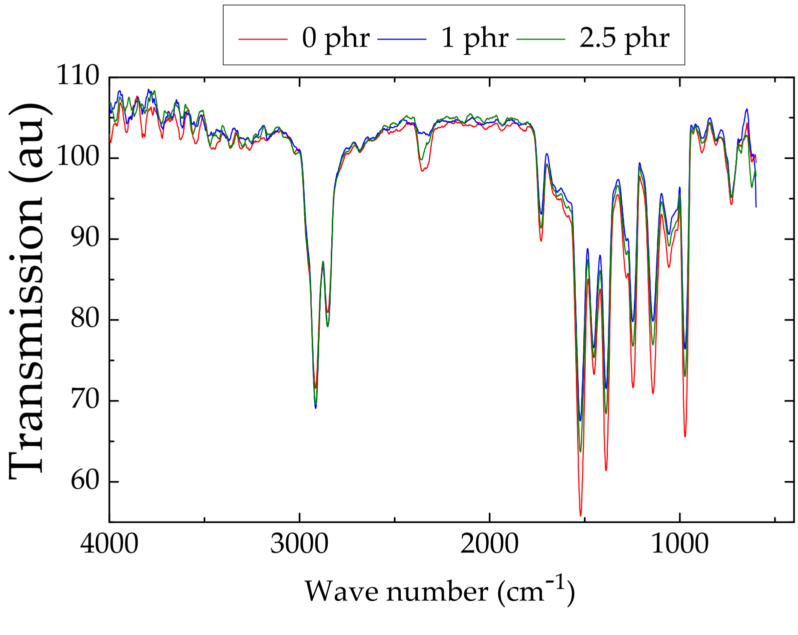

Figure S2. The FTIR spectra recorded on EPDM/$BaTiO_3$ composites exposed to a γ-dose of 100 kGy.
